# Electronic Nursing Records: Importance for Nursing and Benefits of Implementation in Health Information Systems—A Scoping Review

**DOI:** 10.3390/nursrep14040262

**Published:** 2024-11-18

**Authors:** Daniela Ivova Taneva, Vasilka Todorova Gyurova-Kancheva, Angelina Georgieva Kirkova-Bogdanova, Diana Angelova Paskaleva, Yovka Tinkova Zlatanova

**Affiliations:** 1 Department of Nursing Care, Faculty of Public Health, Medical University–Plovdiv, 4004 Plovdiv, Bulgaria; daniela.taneva@mu-plovdiv.bg (D.I.T.); diana.paskaleva@mu-plovdiv.bg (D.A.P.);; 2 Department of Medical Informatics, Biostatistics and Elearning, Faculty of Public Health, Medical University–Plovdiv, 4002 Plovdiv, Bulgaria; angelina.kirkova@mu-plovdiv.bg

**Keywords:** electronic nursing record, electronic health record, nursing care, nursing practice, nursing documentation

## Abstract

**Introduction**: The advancement of nursing science and practice necessitates the documentation of information, which is increasingly being recorded in electronic mediums due to the progress of information technology. Various countries around the world have implemented electronic nursing records (ENRs) or are in the process of implementing them. This study aims to ascertain the significance of electronic nursing records and consolidate their primary benefits for nursing. **Methods**: The study utilized an established scoping review methodology (Arksey and O‘Malley protocol; JBI method; PRISMA ScR (2018)). **Results**: Out of 6970 initial articles extracted from four databases, 36 were included in the study. Several essential elements for structuring, introducing, and emphasizing the importance of ENRs have been recognized, including the availability of standardized terminology, enhancement of nursing care quality, advancement of research activity, integration with electronic systems, optimization of healthcare, and conditions for ENR integration. **Conclusions**: Electronic nursing records are indispensable and beneficial for enhancing care quality, improving patient safety, and affirming the autonomy of the nursing profession.

## 1. Introduction

The evolution of nursing from a vocation to a scientific field is closely linked to the emergence of new areas like nursing methodology, nursing classifications, and the nursing process. These advancements necessitate the development of precise and specialized terminology that can ideally be standardized for comprehension by a wide range of nurses. Furthermore, the evolution of the nursing process and terminology naturally results in the need to document gathered evidence to achieve common objectives such as archiving, statistical analysis, research, and enhancement. Nursing documentation has traversed a long and challenging path, evolving from non-existence to paper-based records and now to the digital age with standardized care plans created automatically by purpose-built software. Different countries are at different stages of this progression, and some have yet to fully embrace information and communication technologies. The objective of digitalization is not only to streamline numerous documentation processes but also to enhance and advance the fundamental essence of nursing, namely tailored patient care.

In the 1970s, the WHO recognized new technologies as essential tools for transferring and analyzing information to support the management of healthcare systems [1]. Since then, numerous countries have focused on organizing clinical information to enable patient-centered care and statistical analysis. However, medical documentation often lacks a clear separation between nursing interventions and the contributions of other healthcare professionals. The introduction of electronic health records, which provide medical, administrative, and other data, has highlighted the need to define the content of nursing records within these systems.

There are several examples in Western Europe of nursing algorithms being taken to a higher level, offering standardization, management, scaling, statistics, prediction, and quality management. The rapid development of information technologies has facilitated this progress, integrating digitization into all aspects of life. Digitization enables the creation and standardization of care plans and their consolidation into a nursing file. For instance, the information program Gacela-HIS (formerly Gacela Care) has been pivotal in Spanish healthcare since 1996 [2]. Developed and implemented step by step in collaboration with universities, nurse practitioners, hospital care facilities, and the Ministry of Health and its territorial divisions in the various autonomous regions of the Kingdom of Spain, Gacela-HIS streamlines various functions such as patient and bed availability, work plan scheduling, real-time patient condition monitoring, and the creation of individual nursing care plans [3]. Other software employed in Spanish healthcare, including Selene, OMI-AP, and AP-Madrid, incorporates modules for describing and recording nursing activities. Selene, from Siemens, embodies a comprehensive management approach to healthcare services, encompassing all patient interactions within the healthcare system [4]. The university environment also plays a crucial role in the integration of such software into clinical practice, as demonstrated by the implementation of WiseNurse, aimed at facilitating students’ transition to work and the application of nursing diagnoses [5]. The ongoing trend focuses on enhancing information programs related to nursing diagnosis to select appropriate interventions and improve the quality of care. The introduction of electronic nursing files has the potential to streamline work processes, track activities for evaluation, standardize nursing care, eliminate paper-based practices, provide data for attestation and quality assessment, offer resources for research, distribute tasks among nurses, and enhance overall activities. These software tools aim to consolidate input data to generate diagnostic hypotheses, define characteristics, and connect factors [6]. Furthermore, the pursuit of evidence-based practice in medical and nursing domains emphasizes the significance of standardizing care, making such patterns more attainable.

Nurses and midwives constitute the largest portion of the global healthcare workforce and should utilize standardized terminology in electronic health records (EHRs) [7]. To establish uniform and formalized nursing terminology and taxonomy, many countries utilize nursing classification systems. Unlike the International Classification of Diseases (ICD), they are not universal and each country chooses its own classification. However, most developed countries have adopted a nursing process model that involves nursing diagnoses, which are regularly reviewed every two years with the input of thousands of nurse practitioners from 32 countries [8]. Nursing diagnoses convey professional judgments made by nurses on a daily basis, defining nursing knowledge. Their application enhances nursing practice and establishes consistent standards for nursing care quality, which can then be validated. NANDA-I, in conjunction with the NOC (Nursing Outcomes Classification) and NIC (Nursing Interventions Classification), aims to develop and implement standardized terminology to ensure evidence-based care, enable reimbursement in hospital and outpatient settings, and enhance the quality of nursing care. Although based on functional models, nursing diagnoses in Europe form the foundation of nursing care plans, which are built on the conceptual model of Virginia Henderson, adopted by the International Council of Nurses (ICN) in 1955 (*The Nature of Nursing*) [9]. It is widely considered the most universal conceptual nursing model, despite a lack of data in the literature regarding its implementation process in any specific country, focusing instead on nurses’ attitudes toward different models [10]. A specialized area of nursing science called nursing methodology examines and develops the nursing process using nursing taxonomy.

The present study aims to provide evidence regarding the significance and benefits of implementing electronic nursing records for nursing science and practice.

The study is being carried out as part of an intra-university project focused on applied research, aimed at facilitating the initial implementation of electronic nursing records within the framework of electronic health records at a university hospital in Bulgaria.

## 2. Materials and Methods

We conducted a scoping review using DECS-MeSH descriptors and Boolean operators; the Arksey and O’Malley protocol [11], the Joanne Briggs Institute (JBI) method [12], and the PRISMA flow chart of the Preferred Information Elements for Systematic Reviews and Meta-Analyses [13].

### 2.1. Search Strategy

Two contributors (VG-K and DT) conducted a preliminary search in various databases, including PubMed, EBSCO, and WoS, to define and refine the search strategy and group conceptions. Then, we used four databases as sources: Medline (PubMed), Scopus, Google Scholar, and the Cochrane Library. Inclusion and exclusion criteria were applied:

- Inclusion criteria: Articles in both English and Spanish were included in the study, identified by the researchers as the most utilized languages in medical literature worldwide. The timeframe was constrained to the last 20 years (2004–2024), a decision justified by the advancements in information technologies and their application in healthcare. This conclusion was drawn from preliminary data extraction, in which the average publication time of sources relevant to similar topics was calculated. There were no restrictions based on the type of study; articles were considered irrespective of the research methodologies employed. Additionally, specific inclusion criteria were defined for secondary analysis, focusing on the impact of electronic nursing records (ENRs) on healthcare quality, on the knowledge and skills of nurses, and on the effectiveness of the nursing process. In the subsequent stage, filters were applied to allow access only to articles that were available in full text and offered free of charge.
- Exclusion criteria: languages other than English and Spanish; publications before 2004.

The review followed the criteria outlined by Arksey and O’Malley [11]:

Phase 1: Identification of the research question, and clarification of team members’ roles. The main aim of the research was defined, namely, to provide evidence for the benefits of ENRs for nursing practice.

Phase 2: Identification of relevant studies. The inclusion criteria for the scoping review (article type, time frame, language of publications, specific relevance criteria, full text, and open access) were defined, as well as the exclusion criteria (languages other than those included and publications before 2004). The databases were selected because of the possibility of accessing full texts with open access. A virtual library was used to organize the data (Zotero 7.0.2). DECS-MeSH descriptors were defined based on keywords. Table 1 illustrates the strategy implemented between 16 and 17 August 2024, using Boolean operators and DECS-MeSH descriptors.

**Figure 1:**
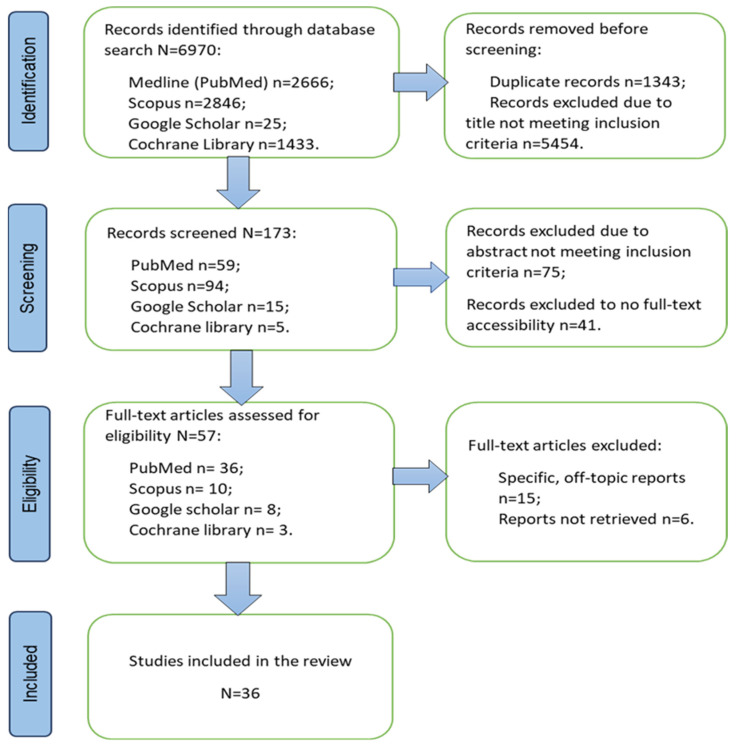
The PRISMA flow chart.

Phase 3: Selection of studies taking into account the inclusion and exclusion criteria. Based on the defined criteria and the aim of the study, the selection of publications to be included in the scoping review according to the PRISMA ScR Checklist requirements began. Three researchers, divided into two groups, participated in the reading of abstracts and full texts, and two acted as referees for the arbitration of discrepancies. The data from the search strategy are presented in Figure 1.

Phase 4: Registration and presentation of data—extraction of data through qualitative analyses (described in detail in data analysis). A joint discussion to develop an analysis framework was carried out. The preliminary results were discussed in accordance with the purpose of the study and their registration and graphical presentation were initiated.

Phase 5: Compilation, summary, and communication of the results—a content analysis was performed on the end list of publications to extract data to summarize the results and answer the research questions regarding the significance of ENR. The results of the analysis are presented in Table 2.

### 2.2. Data Analysis

The preliminary search led to the selection of articles, to which a qualitative thematic analysis was applied to identify main themes that generated ideas for the research questions, namely what the meaning of ENRs is and how it affects various parameters of nursing care and healthcare.

The initial, or “identification”, phase involved selecting manuscripts based on specific filters such as years of publication, the language of the studies, and keywords. The results were obtained by assessing titles, abstracts, and keywords. Given the large volume of literature reviewed, a deductive method of content analysis was applied to group the different ideas into categories (for example, dividing the publications into groups concerning the impact of ENRs on the quality of care or user satisfaction). The second phase entailed conducting a full-text review to exclude any articles that did not meet the specified criteria. An inductive approach to content analysis was used, which led to the identification of more specific concepts related to ENRs—the possibility of working with standardized nursing classifications; the effect of ENRs on the quality of care; increased research due to the use of ENRs; the impact of ENRs on users; efficiency of health processes thanks to ENRs; and identification of optimal conditions for the implementation of ENRs. The following information was extracted from each article: database, author/title/year, country and/or city, type of study, the objective of the study, and main conclusions or focal points.

## 3. Results

The initial search yielded a total of 6970 articles from four databases. Due to the broad inclusion criteria, the collaborative efforts of three authors (VG-K, DT, and AK-B) were necessary to screen the results. As a result, duplicates and those whose titles did not correspond to the subject matter were removed. From PubMed, 583 duplicates and 2023 mismatches were removed; from Scopus—433 duplicates and 2307 mismatches; from Google Scholar—1 repeat and 9 not responding; and from the Cochrane Library, 313 duplicates and 1115 not responding. In the screening phase, 173 publications were processed, 41 of which we did not have free access to the full text despite the specified criteria. Another 75 studies did not meet the specific criteria set in their abstracts—the impact of ENRs on the quality of health care, the knowledge and skills of nurses, and the effectiveness of the nursing process. In the process of reviewing the articles for eligibility, 57 were selected, 21 of which were eliminated due to topics unrelated to the research questions or those that could not be extracted in the full text. The results of the search are presented through the PRISMA flow chart in Figure 1.

Following the search, all identified articles were imported into Zotero 7.0.2 (Digital Scholar, Vienna, VA, USA). This scoping review included 36 original papers, scoping reviews, literature and systematic reviews, as well as randomized and quasi-experimental trials. Generally, qualitative studies predominate. Table 2 outlines the primary characteristics of the chosen articles.

## 4. Discussion

This scoping review focuses on key topics relevant to the successful development and integration of standardized nursing electronic documentation within health information systems to positively impact nursing practice and theory and emphasize the significance of nursing care.

### 4.1. Ability to Work with Standardized Nursing Information Regarding Nursing Classifications

Several studies have highlighted the critical need for an appropriate taxonomy that not only supports the nursing process but also communication between individual units. In addition, from a theoretical perspective, nursing classifications establish and differentiate nursing terminology and language from that of other medical professionals. As noted by Hannah et al. [16], when discussing the situation in Canada (replicable in other countries), the focus is primarily on the information needs of physicians. Despite nurses being the largest group of care providers in the Canadian healthcare system, the care they provide has a significant impact on clinical patient outcomes and yet remains largely invisible in most EHRs [16]. There are several classification systems currently in practice that aim to cover the essence of the nursing process on a broad scale. These systems involve the collection, analysis, and validation of information; making a nursing diagnosis; planning appropriate care and focusing on expected outcomes; implementing interventions, and evaluating the process. These systems are well-suited to operate in an electronic environment and serve as the basic building blocks of electronic nursing records (ENRs). Standardizing these systems can optimize the processes related to ENRs and enhance the quality of patient care. In their study, Heidarizadeh et al. [17] aim to demonstrate the effectiveness of standardization through the use of the CCC (Clinical Care Classification) system, concluding that CCC is a suitable method for standardizing nursing reports and enhancing the quality of electronic nursing records from a structural perspective. In addition to CCC, other systems such as the International Classification of Nursing Practice (ICNP), the Omaha System, and NANDA are commonly used examples of standardized nursing terminology. International research shows that the implementation and use of nursing standards in EHRs can increase the ability to distinguish, extract, and analyze nursing care to improve quality and safety, including improvements in nurses’ knowledge of evidence-based clinical guidelines [25]. A study in Norwegian practice showed that standardized care plans led to a reduction in administrative burden, improved documentation quality, and enabled the identification of patient care needs and more effective management of long-term conditions [25]. A series of conferences in Nashville, Tennessee (USA) over the past 25 years has aimed to model and develop a standard nursing terminology that can be successfully integrated into reference information models such as HL7. The process of building the Nursing Information Reference Model (NIRM) as a standard for entering electronic nursing information within HL7 is outlined in the study by Goossen et al. [38]. The purpose of the study by Brazilian researchers was to examine the relationship between data and information in the nursing process, which were computerized according to ICNP^®^ version 1.0, and to establish relationships between detailed clinical assessments of each human system and nursing diagnoses, interventions, and outcomes [45]. Evolutionarily, the process of standardizing nursing care is lengthy and labor-intensive due to the wide array of nursing knowledge and practices across different regions of the world. There is a high priority on the use of interoperable electronic health records to support data exchange between information systems and generate secondary data for research [47]. A later study on the same topic and with the same lead collaborator (Westra et al.) [48] tracked over ten years of work on the USA National Action Plan for shared and comparable nursing data to support practice and translational research to transform healthcare. In another study by Müller-Staub et al. [49], the aim was to investigate the effect of directed clinical reasoning as a teaching method to promote nurses’ abilities to use standardized language.

### 4.2. Improving Health Management, Increasing the Quality of Care and Reducing the Risk of Errors

Besides the aforementioned advantages of ENR, its main direction and prerequisite for its introduction is the improvement of patient-centered care, where efficiency, effectiveness, safety, and quality are emphasized. Several studies have aimed to demonstrate the proportional relationship between the use of electronic records and the improvement of health care. In a prospective study, Zhang et al. [15] describe the potential for developing EHR quality control systems, which have a direct impact on the quality of care documented in electronic records. In their retrospective study, Chang et al. [32] analyzed data from quality audit software. The study was implemented to assess the status of EHR in a hospital in Taiwan with the aim of reducing errors. A pilot study among nurses in Israel on the effectiveness of EHRs in relation to errors, workload, and availability of medical information yielded conflicting results in several directions and set the stage for future studies [26]. Jayousi et al. [28] shared the experiences of Italian nurses regarding their work with information technology and documented improvements in the quality of care, optimization of health management, and professional satisfaction. A literature review by other Italian authors examines how the introduction of nursing data into electronic documents reduces the dispersion of information, promotes its sharing with other health professionals, and contributes to multidisciplinary care management, leading to a better formulation of multidisciplinary therapeutic and health-educational plans [30].

### 4.3. ENRs as a Prerequisite for Scientific Research

The information, generated electronically, is available in a database, which is a prerequisite for statistical processing and a large set of useful information to be selected and summarized in different types of scientific studies in the field of nursing. In general, all kinds of methods are needed to stimulate and improve scientific research in the field of nursing practice, and EHRs and ENRs are suitable sources for this. A literature review by Luan et al. [29] demonstrates the growing interest in research on electronic nursing records, their use and related elements, and the data they accumulate. Dionisi et al. [30], in addition to the above-mentioned advantages for nursing practice and health care, prove the existence of ENRs as a prerequisite for the development of scientific research activity.

### 4.4. Impact of Electronic Records on the Behavior, Attitudes, and Knowledge of Medical Professionals/Students

Most research has been conducted in the area of qualitative and quantitative measurements of perceptions and attitudes of nurses, other health professionals, and students towards the use of electronic medical records. The reason for this is the desire to achieve improvements in the training process, the implementation, and the very use of information technologies related to health care. Thanks to these studies, new strategies have been consolidated, including relevant policy decisions, training programs at the university or hospital level, redistribution of resources, and adaptation of new technologies. All these steps aim to overcome the change barriers (if any) to the implementation of electronic records and the implementation of the plan for all electronic health care. Many countries are yet to introduce similar types of resources into their health care systems, as is the case of Ethiopia, and a natural consequence of the process is a study of the willingness of health workers to use EHR and the factors that influence it [14]. Studies with similar objectives yield different results depending on cultural differences, the level of health education, and some personality characteristics. A study by Ahn et al. [18] deals with the timeliness of entering information into the EHR and summarized that it depends on various factors, such as the professional experience of the nurse. Other countries with more experience in electronic records are conducting studies on whether the elements of EHRs meet the needs of users. Such is the case with the study by Stevenson et al. [19], which in 2010 concluded that Swedish nurses were not sufficiently satisfied with EHRs because they did not reflect their functions. Such studies are a prerequisite for the improvement of the relevant electronic systems and the introduction of nursing modules, ENR, and the like within information health systems. Brazil also introduced ENRs relatively early in specific departments and focused efforts on improving worker perceptions of the new documentation system. The results are divided into three categories: favorable and unfavorable aspects of the introduction, as well as expectations of the process itself [20]. On the other hand, there are studies on specific elements of the ENR, such as the nursing report. Meum et al. [21] found that a greater share of the studied sample was satisfied with delivering nursing information this way. Another pioneer country such as the USA has already been working on the usability of EHRs and ENRs in 2009, demonstrating variation depending on the user interface with a constant drive for improvement [22]. In general, usability and functionality depend mainly on time efficiency for data entry and intuitiveness of systems, which facilitates the work of increasingly busy nurses. Another essential factor in the utilization of electronic documentation systems is the level of training of the users. This is also proven by a Norwegian study by Laukvik et al. [25] involving nurses and Irwin et al. [27] with students in Australia. It is important to note that there are contrasting reports, in which, for some of the participants, one feature of EHR is a disadvantage, and for others, it is an advantage, as demonstrated in a study by Naamneh et al. [26]. In some works, such as the case described in the article by Topaz et al. [33], negative results are mentioned already in the title. The study is of considerable value, given its scale (45 countries and over 450 respondents), and defines the EHR problems perceived by medical professionals at that stage (2016) [33]. Despite the prevailing negative attitude towards electronic documentation, this type of research is positively focused on processes of improvement, correction, and modification. Several studies aimed to identify those factors that determine nurses’ attitudes toward information systems. In the UK, Winn et al. [39] attempt to demonstrate a relationship between demographic and other factors and attitudes toward electronic documentation based on the Unified Theory of Acceptance and Use of Technology (UTAUT). Some of these factors are gender, age, experience, voluntariness, and appropriate conditions of use, as shown in the study by Alrasheeday et al. [41] in Saudi Arabia. In summary, the younger age of users, previous experience with information technology, and higher educational level are key factors in fostering a positive attitude towards EHR. Regarding technological skills, a Finnish study focused only on this factor, resulting in different levels of technological competence among users, which in turn led to a study of attitudes toward health information systems [40]. Proportionality between high technological competence and positive evaluations of the systems is proven [40].

Identifying those specific elements of electronic health records that do or do not function is at the core of the quest for many researchers. Depending on the country and the electronic system that is used, different components are assessed. However, nursing functions are similar and the usability of electronic documentation depends on workload, knowledge and skills, habits, and preferences. Lloyd et al. [43] surveyed nurses and physicians in Australia to find out exactly which items were rated as functional and useful and which were not. A similar approach was taken at the University of Tabriz (Iran) to evaluate implemented nursing software [46]. Despite the obtained results, their subjectivity should be taken into account, bearing in mind that technological evaluations require technological knowledge and experience. This is the case described in a study by Khudhayer Al-Aubaidy et al. [42], demonstrating the low level of knowledge about EHRs in Iraq regardless of demographic differences or educational level.

### 4.5. Automation and Optimization of Processes

The concept of electronic healthcare revolves around optimizing services for better quality. Even new technologies like ENRs can be improved. For instance, additional software can audit and optimize record quality, reducing input time, minimizing errors, and increasing satisfaction [15]. Traditional nursing duties, like nursing reports, have also been restructured for optimization. Norwegian hospitals achieved a major breakthrough in this area 15 years ago, emphasizing integrating new technologies into established processes [21].

Hyun et al. [22] emphasize the connection between nursing practice and supporting systems, highlighting the need for studies aimed at automating all records. Such a study is that of Rouleau et al. [24], in which the authors present a system for ENR optimization within the so-called “nursing effectiveness framework”. The stages are bilaterally oriented and include material/human resources and their management; converting resources into services; and services produce specific changes in patients’ conditions [24].

Various studies on EHR/ENR optimization have involved users to improve electronic documentation. For example, a 2022 Canadian study engaged nurses to identify interventions for EHR redesigns [44]. In a study by Drnovšek et al. [36], students compare two information systems in Slovenian healthcare, aiming to encourage users’ participation in developing future solutions. The Iranian nursing-oriented software, developed based on a literature review, addresses historically proven errors. The team emphasizes the meaningful involvement of end users during the development and implementation process as a critical condition for success [37].

### 4.6. Conditions for Implementation of Electronic Nursing Documentation

Progress is a one-way phenomenon and requires movement in the right direction. In the case of electronic documentation, nursing care should be an integral and mandatory part of it, serving to identify the role and place of the nurse in the process of patient care. Creating optimal conditions for the implementation of ENRs is not an easy task and requires the participation of numerous institutions and the involvement of nurses themselves. Although the process of introducing W. Henderson’s conceptual model in many countries has not been described anywhere, except for sentiments towards it [10], the implantation of ENRs as a process in the new millennium has been documented in detail, something of great benefit to health systems that they have not accepted it yet. An example of this is a study by Kleib et al. [34], which reflects the users’ preliminary evaluation of a specific nursing file with educational purpose in undergraduate programs. The pilot study aims to detect feasibility issues for the implementation of the program and includes the views of students and faculty regarding the software itself [34]. The progress of ESD implementation in a South Korean university hospital and the benefits for nursing practice have been described in detail [23]. The specific software has a terminology server and a nursing documentation system, which demonstrates the importance of a technological resource for successful integration [23]. The aforementioned study by Strudwick et al. [44] used a three-phase scheme to specify the events that influence the effectiveness of the electronic documentation implementation processes: (1) determination and validation of the key indicators for nursing documentation; (2) identification of EHR usage trends and areas for improvement; and (3) the creation of ideas and their implantation through the necessary technological support. Another case is Brazil, which since 2010 has been working on the development and implementation of a computerized system reflecting the nursing process based on the international nursing standard ICNP [45]. Although at the time they recognized the applicability of the software only for the work of intensive care units, they emphasized the possibility of creating conditions for expanding the field of application in other structures and for more nurses [45]. The development of the integration of nursing standards in US EHRs is the subject of the work of Westra et al. [48], in which, in addition to standardization, the conditions that led to this implementation are visible, namely knowledge, practice, policies/resources, and research activity. In the late 20th and early 21st centuries, Canadian healthcare identified the need to implement electronic nursing documentation, which received significant support from the Canadian nursing community and other key healthcare leaders in each participating province [16]. The interpretation of these circumstances visualizes another necessary condition for implantation, namely unity. Another condition is described in the work of Bjarnadottir et al. [35] tracking EHR integration in USA nursing homes. With a representative sample of over 900 of these medical and social institutions, the conclusion was reached that targeted policy and support for change are needed. Countries with less experience, such as Iran, take advantage of the errors and shortcomings reported in the literature to build and implement their own nursing module with improved parameters, emphasizing the importance of the participation of future users in these processes [37].

## 5. Conclusions

The nursing profession and its role and place in healthcare are in a process of constant development, which requires the incorporation of criteria to confirm its autonomy. The same applies to nursing science, which develops in parallel with medical science and with the evolution of technologies. Bridges are needed between nursing models and information healthcare.

The American Nursing Association (ANA) emphasizes the importance of standardized terminologies as a significant means of facilitating interoperability between different concepts, nomenclatures, and information systems [50].

In the era of universal digitization of healthcare, it is crucial for nurses to master electronic documentation systems in order to provide effective patient care [51]. The findings of this scoping review help in understanding the benefits and advantages of electronic documentation in improving nursing practice. It is important to emphasize the necessity of electronic nursing records, along with the essential elements and conditions required for their proper structuring, introduction, and use, and the need for regular updating and upgrading. Most studies highlight advantages such as enhancing theoretical knowledge and working with standardized nursing care plans, improving care management and coordination, increasing patient safety, streamlining administrative processes, and enhancing intra- and interdisciplinary communication.

Systematization can serve as a framework for medical institutions, universities, and politicians to effectively organize, monitor, and enhance the integration of electronic nursing documentation for improved patient care and healthcare management. We recommend conducting future systematic reviews to gather more detailed information on the issues addressed in this study.

## Figures and Tables

**Table 1:** Search strategies in the different databases.

Database	Search Strategy	Search Date
Medline (PubMed)	(nursing records) OR (electronic health records) AND (nursing care)	16 August 2024
Scopus	(nursing records) OR (electronic health records) AND (nursing care)	16 August 2024
Google Scholar	nursing electronic records	17 August 2024
Cochrane Library	nursing AND electronic AND records	17 August 2024

**Table 2:** Analysis of the articles included in the scoping review ^1^.

	Database	Authors/Title/Year	Country/ City	Type of Study	Research Focus	Main Results/Highlights
1	PubMed	Ngusie, H.S., Kassie, S.Y., Zemariam, A.B. et al. Understanding the predictors of health professionals’ intention to use electronic health record system: extend and apply UTAUT3 model. 2024 [14]	Ethiopia	Statistical qualitative	To reveal and understand predictors of healthcare professionals’ intention to use electronic health records (EHRs).	Readiness to use EHRs depends on personal initiative, motivation, fear of technology, and social variables with a distinct gender difference.
2	PubMed	Zhang, S., Quan, Y.Y. and Chen, J. Construction and application of an ICU nursing electronic medical record quality control system in a Chinese tertiary hospital: a prospective controlled trial. 2024 [15]	China	A prospective controlled study	Development and evaluation of an EHR quality control system in an emergency department targeting electronic nursing records (ENRs).	Electronic medical record quality control systems application increases efficiency, reduces the risk of errors, and ensures patient safety and quality of care.
3	PubMed	Hannah KJ, White PA, Nagle LM, Pringle DM. Standardizing nursing information in Canada for inclusion in electronic health records: C-HOBIC. 2009 [16]	Canada	-	Standardization of nursing information visualized in EHRs at the national level and trial of a system adapted to this.	The introduction of a specific ENR model within EHRs supports the standardization of nursing care and processes. It is well accepted by the users.
4	PubMed	Heidarizadeh K, Rassouli M, Manoochehri H, Tafreshi MZ, Ghorbanpour RK. Effect of electronic report writing on the quality of nursing report recording. 2017 [17]	Iran	A quasi-experimental study	Determining the effect of ENR use on the quality of nursing documentation.	The introduction of a standardized language for nursing records (in this case the Clinical Care Classification (CCC) system) leads to an increase in the quality of nursing records.
5	PubMed	Ahn M, Choi M, Kim Y. Factors Associated with the Timeliness of Electronic Nursing Documentation. 2016 [18]	South Korea	A retrospective quantitative study	A study of factors associated with timely entry of nursing information into EHRs.	The timeliness of data entry depends on the nurse’s experience, day of the week, and work shift.
6	PubMed	Stevenson JE, Nilsson GC, Petersson GI, Johansson PE. Nurses’ experience of using electronic patient records in everyday practice in acute/inpatient ward settings: A literature review. 2010 [19]	Sweden	Literature review	Research of nurses’ views on the use of EPRs.	The use of EHRs and BIS in many cases is not designed by/for nurses and they experience difficulties in entering information.
7	PubMed	Lima AF, de Oliveira Melo T. Percepção de enfermeiros em relação à implementação da informatização da documentação clínica de enfermagem [Nurses’ perceptions regarding the implementation of computer-based clinical nursing documentation]. 2012 [20]	Brazil	A descriptive qualitative study	An analysis of nurses’ perceptions of the implementation of computer-based clinical nursing documentation in surgical units.	Nurses support the module’s introduction due to training strategies and suggestion opportunities.
8	PubMed	Meum T, Wangensteen G, Soleng KS, Wynn R. How does nursing staff perceive the use of electronic handover reports? A questionnaire-based study. 2011 [21]	Norway	Analytical qualitative research	Assessing attitudes and perceptions about electronic nursing reporting in a university hospital.	Most of the working nurses are satisfied with the electronic reports.
9	PubMed	Hyun S, Johnson SB, Stetson PD, Bakken S. Development and evaluation of nursing user interface screens using multiple methods. 2009 [22]	USA, New York	-	Investigating nurses’ perceptions of ENR functionality and creating user interface screens.	The effectiveness of BIS and EHRs depends on their modeling to fulfill the needs of users.
10	PubMed	Min YH, Park HA, Chung E, Lee H. Implementation of a next-generation electronic nursing records system based on detailed clinical models and integration of clinical practice guidelines. 2013 [23]	South Korea	-	Defining the components of a next-generation ENR system providing full semantic interoperability and evidence integration into the nursing record system.	The results consist of the successful implementation of ENRs in a hospital in Seoul based on clinical models and clinical practice guidelines. It is a prerequisite for successfully maintaining nursing practice and documentation.
11	PubMed	Rouleau G, Gagnon M, Côté J, Payne-Gagnon J, Hudson E, Dubois C Impact of Information and Communication Technologies on Nursing Care: Results of an Overview of Systematic Reviews. 2017 [24]	Canada	Overview of systematic reviews	Analysis of dimensions and indicators of nursing care potentially influenced by information technology.	There are aspects of the nursing process that are affected by various information and communication technologies and can be optimized.
12	PubMed	Laukvik LB, Lyngstad M, Rotegård AK, Fossum M. Utilizing nursing standards in electronic health records: A descriptive qualitative study. 2024 [25]	Norway	A descriptive qualitative study	Analysis of the experience and the perceptions of nurses, working in nursing homes regarding ENR use and standardized taxonomy.	ENR knowledge and skills affect the quality of records; the organization of ENRs influences the motivation to work; usability issues impede normal workflow; ENR standardization leads to improved practice and advances nursing knowledge.
13	PubMed	Naamneh, R., Bodas, M. The effect of electronic medical records on medication errors, workload, and medical information availability among qualified nurses in Israel—a cross sectional study. 2024 [26]	Izrael	A descriptive cross-sectional study	Research of the position of medical staff regarding the impact of EMR systems on factors related to patient safety, including medication errors, workload, and availability of medical information.	The results indicate a positive attitude towards EHRs regarding safety enhancement but indicate deficiencies regarding the ability to input the information.
14	PubMed	Irwin P, Hanson M, McDonald S, Noble D, Mollart L. Nursing students’ perspectives on being work-ready with electronic medical records: Intersections of rurality and health workforce capacity. 2024 [27]	Australia	Online survey/qualitative research	A survey of student nurses’ views on preparedness for EHR use.	Students feel more confident using EHRs during their training process and consider it necessary to introduce simulation programs for better clinical work after graduation.
15	PubMed	Jayousi S, Barchielli C, Alaimo M, Caputo S, Paffetti M, Zoppi P, Mucchi L. ICT in Nursing and Patient Healthcare Management: Scoping Review and Case Studies. 2024 [28]	Italy	Scoping review/case studies	Exploration of a wide range of information and communication technologies used in nursing and healthcare.	This article highlights how these technologies have improved the efficiency, accuracy, and accessibility of clinical information, contributing to improved patient care, safety, satisfaction, and management.
16	PubMed	Luan Z, Zhang Z, Gao Y, Du S et al. Electronic health records in nursing from 2000 to 2020: a bibliometric analysis. 2023 [29]	China	Literature review	A survey of the application of EHRs in nursing and identification of the current research status quo on the subject.	Studies on the use of EHR in nursing are increasing every year. They are a prerequisite for the development of collaboration and research trends to improve the use of information and communication technologies.
17	PubMed	Dionisi S, Di Simone E, Alicastro GM, Angelini S, Giannetta N, Iacorossi L, Di Muzio M. Nursing Summary: designing a nursing section in the Electronic Health Record. 2019 [30]	Italy	Literature review	Analyzing the components required to include a nursing section in EHRs.	The introduction of EHRs within the framework of EHRs contributes to the multidisciplinary management of care, improvement of their quality, and expansion of health information and is a prerequisite for scientific research.
18	PubMed	Poissant L, Pereira J, Tamblyn R, Kawasumi Y. The impact of electronic health records on time efficiency of physicians and nurses: a systematic review. 2005 [31]	Canada	A systematic review	Investigation of the impact of EHRs on the times for physician and nurse documentation and identify factors that may explain differences in effectiveness between studies.	Time efficiency is a major factor in the successful implementation and use of EHRs.
19	PubMed	Chang HM, Huang EW, Hou IC, Liu HY, Li FS, Chiou SF. Using a Text Mining Approach to Explore the Recording Quality of a Nursing Record System. 2019 [32]	Taiwan	A retrospective quantitative study	Nursing record system quality analysis using SAS Text Miner software.	The software successfully identifies errors in the nursing record system and can be used as an audit system to assess the quality of nursing records.
20	PubMed	Topaz M, Ronquillo C, Peltonen LM, Pruinelli L, Sarmiento RF, Badger MK, Ali S, Lewis A, Georgsson M, Jeon E, Tayaben JL, Kuo CH, Islam T, Sommer J, Jung H, Eler GJ, Alhuwail D, Lee YL. Nurse Informaticians Report Low Satisfaction and Multi-level Concerns with Electronic Health Records: Results from an International Survey. 2017 [33]	International study	Descriptive cross-sectional	This study presents a qualitative analysis of nurses’ satisfaction and problems with current EHR systems.	The study reveals low satisfaction among nurses regarding the nursing functions in EHRs, and the identified factors for this are problems with the systems, lack of functionality, lack of nursing modules in EHRs, lack of user training, etc.
21	PubMed	Kleib M, Jackman D, Duarte Wisnesky U, Ali S. Academic Electronic Health Records in Undergraduate Nursing Education: Mixed Methods Pilot Study. 2021 [34]	Canada	Survey qualitative research	Preliminary evaluation of the Lippincott DocuCare simulated electronic health record and to determine the feasibility issues associated with its implementation.	Study participants support the nursing module and develop strategies and recommendations for its integration into baccalaureate nursing programs in western Canada.
22	PubMed	Bjarnadottir RI, Herzig CTA, Travers JL, Castle NG, Stone PW. Implementation of Electronic Health Records in US Nursing Homes. 2017 [35]	CAIII USA	A randomized cross-sectional study	Assessing the implementation of electronic health records (EHRs) in US nursing homes to determine the characteristics of introducing homes and assess their impact on service quality.	The adoption of EHRs in US nursing homes is moving at a slower pace than in active care hospital settings. However, research indicates that the quality of care has demonstrated improvement following the implementation of EHR systems.
23	PubMed	Drnovšek R, Milavec Kapun M, Rajkovič V, Rajkovič U. Analysis of Two Diverse Nursing Records Applications: Mixed Methods Approach. 2022 [36]	Slovenia	Mixed research (quantitative/qualitative)	A comparison of user experience and perceived quality of nursing process integration in two different electronic nursing care plan documentation applications.	Different results were found regarding elements of nursing care implemented in the two software applications; student perceptions were assessed. The limitation lies in the insufficient experience of the students and the resulting impossibility of objective assessment.
24	PubMed	Shafiee M, Shanbehzadeh M, Nassari Z, Kazemi-Arpanahi H. Development and evaluation of an electronic nursing documentation system. 2022 [37]	Iran	A four-step sequential methodological approach: literature review, Delphi analysis, module construction, evaluation of its use	Process description of the design and content evaluation of the electronic clinical nursing record system.	The minimum acceptable clinical information is entered into the ENR, which reduces the burden of paper documentation and increases user satisfaction.
25	PubMed	Goossen WT, Ozbolt JG, Coenen A, Park HA, Mead C, Ehnfors M, Marin HF. Development of a provisional domain model for the nursing process for use within the Health Level 7 reference information model. 2004 [38]	International study	-	Definition of standardized nursing terminology within the ENR for its inclusion in HIS with HL7.	It is possible to model and map nursing information into the overall healthcare information model. Integrating nursing information, terminology, and processes into information models is a first step toward making nursing information machine-readable in electronic patient records.
26	Scopus	Wynn M, Garwood-Cross L, Vasilica C, Griffiths M, Heaslip V, Phillips N. Digitizing nursing: A theoretical and holistic exploration to understand the adoption and use of digital technologies by nurses. 2023 [39]	UK	Literature review	Examining how key demographics such as gender, age, and voluntary technology use interact to influence nurses’ adoption and use of digital technologies.	Demographic and personality factors influence the integration of ENRs and suggest using individual strategies for success. A holistic approach is necessary to overcome barriers to change.
27	Scopus	Kaihlanen AM, Elovainio M, Virtanen L, Kinnunen UM, Vehko T, Saranto K, Heponiemi T. Nursing informatics competence profiles and perceptions of health information system usefulness among registered nurses: A latent profile analysis. 2023 [40]	Finland	Cross-sectional study	Investigating different profiles of nursing information competencies and their relationship to perceptions of the usefulness of HIS.	Different levels of competence have been identified, with technological competence directly proportional to the positive perception of the usefulness of HISs. Educational strategies for nurses are needed to improve digital health knowledge and skills.
28	Scopus	Alrasheeday AM, Alshammari B, Alkubati SA, Pasay-an E, Albloushi M, Alshammari AM. Nurses’ Attitudes and Factors Affecting Use of Electronic Health Record in Saudi Arabia. 2023 [41]	Saudi Arabia	Cross-sectional study	Assessment of nurses’ attitudes towards EHR and related factors influencing EHR implementation in different hospitals in Saudi Arabia.	Various factors have been identified that influence positive attitudes towards EHR: younger people, with a master’s degree, men and with prior computer experience. Involving nurses in decision-making processes and addressing their concerns can promote favorable attitudes toward EHR implementation.
29	Scopus	Al-Aubaidy, H.F.K.; Abdulwahhab, M.M. (2023). Assessment of nurses’ knowledge toward electronic nursing documentation. Assessment of nurses’ knowledge toward electronic nursing documentation. 2023 [42]	Iraq	A descriptive study	Assessment of nurses’ knowledge about ENR.	Knowledge of electronic nursing records in Iraq is unsatisfactory and unrelated to age, gender, or education level.
30	Scopus	Lloyd S, Long K, Probst Y. et al. Medical and nursing clinician perspectives on the usability of the hospital electronic medical record: A qualitative analysis. 2023 [43]	Australia	Qualitative research	Research of physicians’ and nurses’ opinions about EMR usability.	There is a positive attitude towards the possibility of access from any location, easy documentation of drug therapy, and the possibility of visualizing clinical and instrumental studies. Disadvantages reported are system complexity, difficult communication with primary care, and lack of time resources.
31	Scopus	Strudwick G, Jeffs L, Kemp J. et al. Identifying and adapting interventions to reduce documentation burden and improve nurses’ efficiency in using electronic health record systems (The IDEA Study): protocol for a mixed methods study. 2022 [44]	Canada	A three-phase mixed study	Engaging nurses to generate ideas for supporting and optimizing their experiences with ENR systems, improving efficiency, and reducing ENR-related burdens within organizations.	Understanding the key factors related to inefficiencies in ENRs and overcoming them will reduce the risk of documentation overload for nurses, and facilitate the discovery of methods to improve electronic documentation.
32	Scopus	Dal Sasso GT, Barra DC, Paese F, de Almeida SR, Rios GC, Marinho MM, Debétio MG. Computerized nursing process: methodology to establish associations between clinical assessment, diagnosis, interventions, and outcomes. 2013 [45]	Brazil	A three-stage methodological study	Making a connection between nursing assessment, diagnosis, interventions, and outcomes within ENRs in an emergency department and the ICNP International Classification System.	Standardizing nursing language and taxonomy, as well as establishing logical relationships through an international classification system, enhances nurses’ ability for clinical decision-making, reasoning, and interdisciplinary communication.
33	Google Scholar	Parvan K, Hosseini FA, Jasemi M. et al. Attitude of nursing students following the implementation of comprehensive computer-based nursing process in medical surgical internship: a quasi-experimental study. 2021 [46]	Iran	A quasi-experimental study.	Assessment of nursing students’ attitudes towards ENRs at the University of Tabriz.	Positive ratings for the software being tested have been reported in relation to the prioritization of care, completeness of electronic information, and time-saving. Negative feedback has been received regarding the software’s inability to account for fair distribution of labor and workload.
34	Google Scholar	Westra BL, Delaney CW, Konicek D, Keenan G. Nursing standards to support the electronic health record. 2008 [47]	USA	-	To determine the status and level of nursing standardized terminologies to support the development, exchange, and communication of nursing data.	The standardization of nursing terminology is increasingly important and directly related to the digitalization of health information. Standards in ENRs allow for embedding in EHR, result optimization, and improvement in quality.
35	Google Scholar	Westra BL, Latimer GE, Matney SA, Park JI. et al. A national action plan for sharable and comparable nursing data to support practice and translational research for transforming health care. 2015 [48]	USA	-	An inventory of the historical context of nursing terminologies, challenges in using nursing data for purposes other than documenting care, and a national action plan to implement and use shared and comparable nursing data for quality reporting and translation.	In the USA, a national plan has been implemented to define and integrate standard and shareable nursing data into the national health information system. This process has taken more than ten years and involves the experience and efforts of many organizations.
36	Cochrane library	Müller-Staub M. Preparing nurses to use standardized nursing language in the electronic health record. 2009 [49]	Switzerland	A cluster-randomized experimental trial	Exploring the effect of Guided Clinical Reasoning on the use of standardized nursing language.	A standardized taxonomy in ENRs helps define accurate nursing diagnoses, outcomes, and interventions.

^1^ EHR—electronic health record; EMR—electronic medical record; ENR—electronic nursing record; HIS—hospital (health) information system.

## Public Involvement Statement

There is no public involvement in any aspect of this research.

## Guidelines and Standards Statement

This manuscript was drafted against PRISMA2020.

## References

[1] Mateos-Garcia M.D. Implementación y evaluación de la documentación enfermera en la historia digital: Experiencia en el hospital Virgen de Valme. Proceedings of the X Simposium AENTDE “Lenguaje Enfermero: Identidad, Utilidad y Calidad”.

[2] Rita-Vizoso R. (2017). Cambios en la Practica Asistencial Tras la Adopción del Modelo de Virginia Henderson. Ph.D. Thesis.

[3] Rubio Sevilla J.C., Arribas Espada J.L. Manual Básico del Programa Gacela. Complejo Hospitalario de Toledo. Direccion de Enfermeria. https://studylib.es/doc/3284092/manual-b%C3%A1sico-para-el-uso-del-programa-gacela.

[4] Sanchez Ros N., Regiosa Gago L.F. (2006). Selene. Informatizacion de la historia clínica electrónica: Implicación sobre el proceso de enfermeria. Enferm. Glob..

[5] Sousa E.C.V., Lopes V.O.M., Fereira L.G., Diniz M.C., Froes B.M.N., Sobreira A.B. (2016). The construction and evaluation of new educational software for nursing diagnoses: A randomized controlled trial. Nurse Educ. Today.

[6] Fennely O., Grogan L., Reed A., Hardiker N.R. (2021). Use of standardized terminologies in clinical practice: A scoping review. Int. J. Med. Inform..

[7] Atanasova V. (2012). Electronic Nursing Record. Manag. Educ..

[8] Grove S.K., Gray J.R. (2008). Investigacion en Enfermeria: Desarrollo de la Practica Enfermera Basada en la Evidencia.

[9] Halloran E.J. (1996). Virginia Henderson and her timeless writings. J. Adv. Nurs..

[10] Parra M.L., Ruiz S.S., Rueda G.S., Porras M.D.B., Donaire L.F., Yarnoz A.Z., Sabater D.A., Peláez S.V., Sábado J.T. (2009). Los modelos en la practica asistencial: Visión de los profesionales y estudiantes de enfermeria. Metas Enferm..

[11] Arksey H., O’Malley L. (2005). Scoping Studies: Towards a Methodological Framework. Int. J. Soc. Res. Methodol..

[12] Peters M.D.J., Godfrey C.M., Khalil H., McInerney P., Parker D., Soares C.B. (2015). Guidance for Conducting Systematic Scoping Reviews. Int. J. Evid. Based Healthc..

[13] Page M.J., McKenzie J.E., Bossuyt P.M., Boutron I., Hoffman T.C., Mulrow C.D., Shamseer L., Tetzlaff J.M., Akl E.A., Brennan S.E. (2021). Declaración PRISMA 2020: Una Guía Actualizada Para La Publicación de revisiones Sistemáticas. Rev. Esp. Cardiol..

[14] Ngusie H.S., Kassie S.Y., Zemariam A.B., Walle A.D., Enyew E.B., Kasaye M.D., Seboka B.T., Mengiste S.A. (2024). Understanding the predictors of health professionals’ intention to use electronic health record system: Extend and apply UTAUT3 model. BMC Health Serv. Res..

[15] Zhang S., Quan Y.Y., Chen J. (2024). Construction and application of an ICU nursing electronic medical record quality control system in a Chinese tertiary hospital: A prospective controlled trial. BMC Nurs..

[16] Hannah K.J., White P.A., Nagle L.M., Pringle D.M. (2009). Standardizing nursing information in Canada for inclusion in electronic health records: C-HOBIC. J. Am. Med. Inform. Assoc..

[17] Heidarizadeh K., Rassouli M., Manoochehri H., Tafreshi M.Z., Ghorbanpour R.K. (2017). Effect of electronic report writing on the quality of nursing report recording. Electron. Physician.

[18] Ahn M., Choi M., Kim Y. (2016). Factors Associated with the Timeliness of Electronic Nursing Documentation. Healthc. Inform. Res..

[19] Stevenson J.E., Nilsson G.C., Petersson G.I., Johansson P.E. (2010). Nurses’ experience of using electronic patient records in everyday practice in acute/inpatient ward settings: A literature review. Health Inform. J..

[20] Lima A.F., de Oliveira Melo T. (2012). Percepção de enfermeiros em relação à implementação da informatização da documentação clínica de enfermagem [Nurses’ perception regarding the implementation of computer-based clinical nursing documentation]. Rev. Esc. Enferm. USP.

[21] Meum T., Wangensteen G., Soleng K.S., Wynn R. (2011). How does nursing staff perceive the use of electronic handover reports? A questionnaire-based study. Int. J. Telemed. Appl..

[22] Hyun S., Johnson S.B., Stetson P.D., Bakken S. (2009). Development and evaluation of nursing user interface screens using multiple methods. J. Biomed. Inform..

[23] Min Y.H., Park H.A., Chung E., Lee H. (2013). Implementation of a next-generation electronic nursing records system based on detailed clinical models and integration of clinical practice guidelines. Healthc. Inform. Res..

[24] Rouleau G., Gagnon M., Côté J., Payne-Gagnon J., Hudson E., Dubois C. (2017). Impact of information and communication technologies on nursing care: Results of an overview of systematic reviews. J. Med. Internet Res..

[25] Laukvik L.B., Lyngstad M., Rotegård A.K., Fossum M. (2024). Utilizing nursing standards in electronic health records: A descriptive qualitative study. Int. J. Med. Inform..

[26] Naamneh R., Bodas M. (2024). The effect of electronic medical records on medication errors, workload, and medical information availability among qualified nurses in Israel—A cross sectional study. BMC Nurs..

[27] Irwin P., Hanson M., McDonald S., Noble D., Mollart L. (2024). Nursing students’ perspectives on being work-ready with electronic medical records: Intersections of rurality and health workforce capacity. Nurse Educ. Pract..

[28] Jayousi S., Barchielli C., Alaimo M., Caputo S., Paffetti M., Zoppi P., Mucchi L. (2024). ICT in nursing and patient healthcare management: Scoping review and case studies. Sensors.

[29] Luan Z., Zhang Z., Gao Y., Du S., Wu N., Chen Y., Peng X. (2023). Electronic health records in nursing from 2000 to 2020: A bibliometric analysis. Front. Public Health.

[30] Dionisi S., Di Simone E., Alicastro G.M., Angelini S., Giannetta N., Iacorossi L., Di Muzio M. (2019). Nursing Summary: Designing a nursing section in the Electronic Health Record. Acta Biomed..

[31] Poissant L., Pereira J., Tamblyn R., Kawasumi Y. (2005). The impact of electronic health records on time efficiency of physicians and nurses: A systematic review. J. Am. Med. Inform. Assoc..

[32] Chang H.M., Huang E.W., Hou I.C., Liu H.Y., Li F.S., Chiou S.F. (2019). Using a text mining approach to explore the recording quality of a nursing record system. J. Nurs. Res..

[33] Topaz M., Ronquillo C., Peltonen L.M., Pruinelli L., Sarmiento R.F., Badger M.K., Ali S., Lewis A., Georgsson M., Jeon E. (2017). Nurse informaticians report low satisfaction and multi-level concerns with electronic health records: Results from an international survey. AMIA Annu. Symp. Proc..

[34] Kleib M., Jackman D., Duarte Wisnesky U., Ali S. (2021). Academic electronic health records in undergraduate nursing education: Mixed methods pilot study. JMIR Nurs..

[35] Bjarnadottir R.I., Herzig C.T.A., Travers J.L., Castle N.G., Stone P.W. (2017). Implementation of electronic health records in US nursing homes. Comput. Inform. Nurs..

[36] Drnovšek R., Milavec Kapun M., Rajkovič V., Rajkovič U. (2022). Analysis of two diverse nursing records applications: Mixed methods approach. Zdr. Varst..

[37] Shafiee M., Shanbehzadeh M., Nassari Z., Kazemi-Arpanahi H. (2022). Development and evaluation of an electronic nursing documentation system. BMC Nurs..

[38] Goossen W.T., Ozbolt J.G., Coenen A., Park H.A., Mead C., Ehnfors M., Marin H.F. (2004). Development of a provisional domain model for the nursing process for use within the Health Level 7 reference information model. J. Am. Med. Inform. Assoc..

[39] Wynn M., Garwood-Cross L., Vasilica C., Griffiths M., Heaslip V., Phillips N. (2023). Digitizing nursing: A theoretical and holistic exploration to understand the adoption and use of digital technologies by nurses. J. Adv. Nurs..

[40] Kaihlanen A.M., Elovainio M., Virtanen L., Kinnunen U.M., Vehko T., Saranto K., Heponiemi T. (2023). Nursing informatics competence profiles and perceptions of health information system usefulness among registered nurses: A latent profile analysis. J. Adv. Nurs..

[41] Alrasheeday A.M., Alshammari B., Alkubati S.A., Pasay-an E., Albloushi M., Alshammari A.M. (2023). Nurses’ attitudes and factors affecting use of electronic health record in Saudi Arabia. Healthcare.

[42] Al-Aubaidy H.F.K., Abdulwahhab M.M. (2023). Assessment of nurses’ knowledge toward electronic nursing documentation. Rawal Med. J..

[43] Lloyd S., Long K., Probst Y., Di Donato J., Alvandi A.O., Roach J., Bain C. (2023). Medical and nursing clinician perspectives on the usability of the hospital electronic medical record: A qualitative analysis. Health Inf. Manag..

[44] Strudwick G., Jeffs L., Kemp J., Sequeira L., Lo B., Shen N., Paterson P., Coombe N., Yang L., Ronald K. (2022). Identifying and adapting interventions to reduce documentation burden and improve nurses’ efficiency in using electronic health record systems (The IDEA Study): Protocol for a mixed methods study. BMC Nurs..

[45] Sasso G., Barra D., Paese F., Almeida S., Rios G., Marinho M., Debétio M. (2013). Computerized nursing process: Methodology to establish associations between clinical assessment, diagnosis, interventions, and outcomes. Rev. Esc. Enferm. USP.

[46] Parvan K., Hosseini F.A., Jasemi M., Thomson B. (2021). Attitude of nursing students following the implementation of comprehensive computer-based nursing process in medical surgical internship: A quasi-experimental study. BMC Med. Inform. Decis. Mak..

[47] Westra B.L., Delaney C.W., Konicek D., Keenan G. (2008). Nursing standards to support the electronic health record. Nurs. Outlook.

[48] Westra B.L., Latimer G.E., Matney S.A., Park J.I., Sensmeier J., Simpson R.L., Swanson M.J., Warren J.J., Delaney C.W. (2015). A national action plan for sharable and comparable nursing data to support practice and translational research for transforming health care. J. Am. Med. Inform. Assoc..

[49] Müller-Staub M. (2009). Preparing nurses to use standardized nursing language in the electronic health record. Stud. Health Technol. Inform..

[50] Inclusion of Recognized Terminologies Supporting Nursing Practice Within Electronic Health Records and Other Health Information Technology Solutions. https://www.nursingworld.org/practice-policy/nursing-excellence/official-position-statements/id/Inclusion-of-Recognized-Terminologies-Supporting-Nursing-Practice-within-Electronic-Health-Records/.

[51] Zaman N., Goldberg D.M., Kelly S., Russell R.S., Drye S.L. (2021). The relationship between nurses’ training and perceptions of electronic documentation systems. Nurs. Rep..

